# Nonlinear association of early serum albumin with 28-day ICU mortality in adults with an admission diagnosis of sepsis: a multicenter eICU cohort study

**DOI:** 10.3389/fmed.2026.1954894

**Published:** 2026-09-29

**Authors:** Yue Xu, Shijing Zheng, Zhiqiang Liu, Shili Zhao

**Affiliations:** 1 Department of Nephrology, Yanqing Hospital of Beijing Chinese Medicine Hospital, Beijing, China; 2 Department of Nephrology, Beijing Fengtai Hospital, Capital Medical University, Beijing, China

**Keywords:** eICU collaborative research database, intensive care unit, mortality, prognosis, sepsis, serum albumin

## Abstract

**Background:**

Serum albumin is frequently measured early in intensive care, but its relation to short-term outcome in adults admitted with sepsis has varied across observational studies. We examined the shape and consistency of the association between early serum albumin and 28-day ICU mortality in a multicenter cohort.

**Methods:**

We conducted a retrospective multicenter cohort study using original eICU Collaborative Research Database version 2.0 tables. Adults with admission-diagnosis text containing sepsis were eligible. The exposure was the first valid albumin measurement obtained within 24 h of ICU admission and before ICU discharge; the endpoint was ICU death within 28 days. Hospital-clustered logistic regression and restricted cubic splines were used. Prespecified analyses addressed measurement timing, missing data, selective albumin testing, short stays, repeated admissions, and discharge alive as a competing event.

**Results:**

Among 23,124 adults with an admission diagnosis containing sepsis, 13,634 (59.0%) had an eligible early albumin measurement. The primary cohort included 11,424 ICU stays and 1,161 deaths. In the primary complete-case model (*n* = 10,795; 1,105 deaths), each 1 g/dL higher albumin concentration was associated with lower odds of 28-day ICU mortality (OR 0.54, 95% CI 0.48–0.62). Restricted cubic spline analysis in the same complete-case population showed an overall association and nonlinearity (both *P* < 0.001), with the steepest risk gradient at lower albumin concentrations. Estimates remained directionally consistent in severity-expanded, multiple-imputation, inverse-probability-weighted, short-stay, Cox, and competing-risk analyses.

**Conclusion:**

Early serum albumin was nonlinearly associated with 28-day ICU mortality and may provide complementary information for early risk assessment, particularly at lower concentrations. The clinical utility of particular concentration ranges and the effects of albumin administration require prospective evaluation.

## Introduction

Sepsis remains a major cause of critical illness and death worldwide (1). Serum albumin is measured frequently during ICU admission and has several physiological roles, including maintenance of oncotic pressure, transport of endogenous and exogenous molecules, antioxidant activity, and support of endothelial homeostasis (2–4). During sepsis, the circulating concentration may decline rapidly as inflammation, altered hepatic synthesis, increased vascular permeability, fluid resuscitation, and pre-existing disease act together. An early albumin result may therefore capture information about both the acute illness and the patient's baseline physiological reserve.

Albumin administration has been studied separately in randomized trials of fluid resuscitation. In ALBIOS, albumin plus crystalloid did not improve 28-day or 90-day survival among patients with severe sepsis or septic shock (5). Subsequent trials, systematic reviews, and sepsis guidelines have continued to inform decisions about resuscitation fluid and albumin use (6–9). Those treatment data provide important clinical context for observational studies of albumin concentration, which address the prognostic information carried by a laboratory measurement rather than the effect of administering albumin.

Low albumin concentrations have been associated with mortality in patients with sepsis, although the published studies differ in setting, sampling time, outcome definition, and adjustment for illness severity (10–14). Some studies have used the admission value, whereas others have examined later measurements or changes over time. These differences make it difficult to determine whether the relation is uniform across the concentration range, especially during the initial hours of ICU care when clinical status and treatment are changing rapidly.

We used original eICU Collaborative Research Database tables to examine the association between the first eligible albumin concentration obtained during the first 24 h of ICU care and death during the subsequent 28 days of ICU stay. We also characterized the concentration-response curve and evaluated the consistency of the association across analyses addressing measurement timing, missing data, selective laboratory testing, short ICU stays, repeated admissions, and discharge alive.

## Materials and methods

### Study design and data source

We conducted a retrospective multicenter cohort study using version 2.0 of the eICU Collaborative Research Database (eICU-CRD), which contains de-identified ICU encounters from participating United States hospitals in 2014–2015 (15). The cohort was reconstructed from the original relational tables. The database was accessed through PhysioNet on August 25–26, 2025 after completion of the required credentialing, training, and data-use agreement. The study is reported in accordance with the STROBE statement (16).

Extraction and analysis code, a variable dictionary, run instructions, and aggregate data underlying the submitted tables and figures are provided in the reproducibility archive. Patient-level eICU records are governed by the PhysioNet data-use agreement and are not redistributed.

### Study population, exposure, and outcome

Eligible stays were those of adults whose admission diagnosis text in the admissionDx table contained the term “sepsis”. The available diagnosis text did not permit reliable ascertainment of suspected infection together with an acute change in Sequential Organ Failure Assessment score from baseline (17, 18). We therefore refer to this population as adults with an admission diagnosis containing sepsis.

The exposure was the first valid serum albumin value obtained from ICU admission to 24 h and before ICU discharge. Albumin was recorded in g/dL; values greater than 0 and less than 6 g/dL were eligible (observed range, 0.6–5.0 g/dL). The primary cohort was restricted to ICU stays of at least 24 h so that the exposure could be ascertained over the same interval for every stay. Analyses using 0–6-h, 0–12-h, and 0–48-h windows were prespecified. Cohort selection is shown in Figure 1, and the source fields, units, and derivation rules are described in Supplementary Table 5.

**Figure 1:**
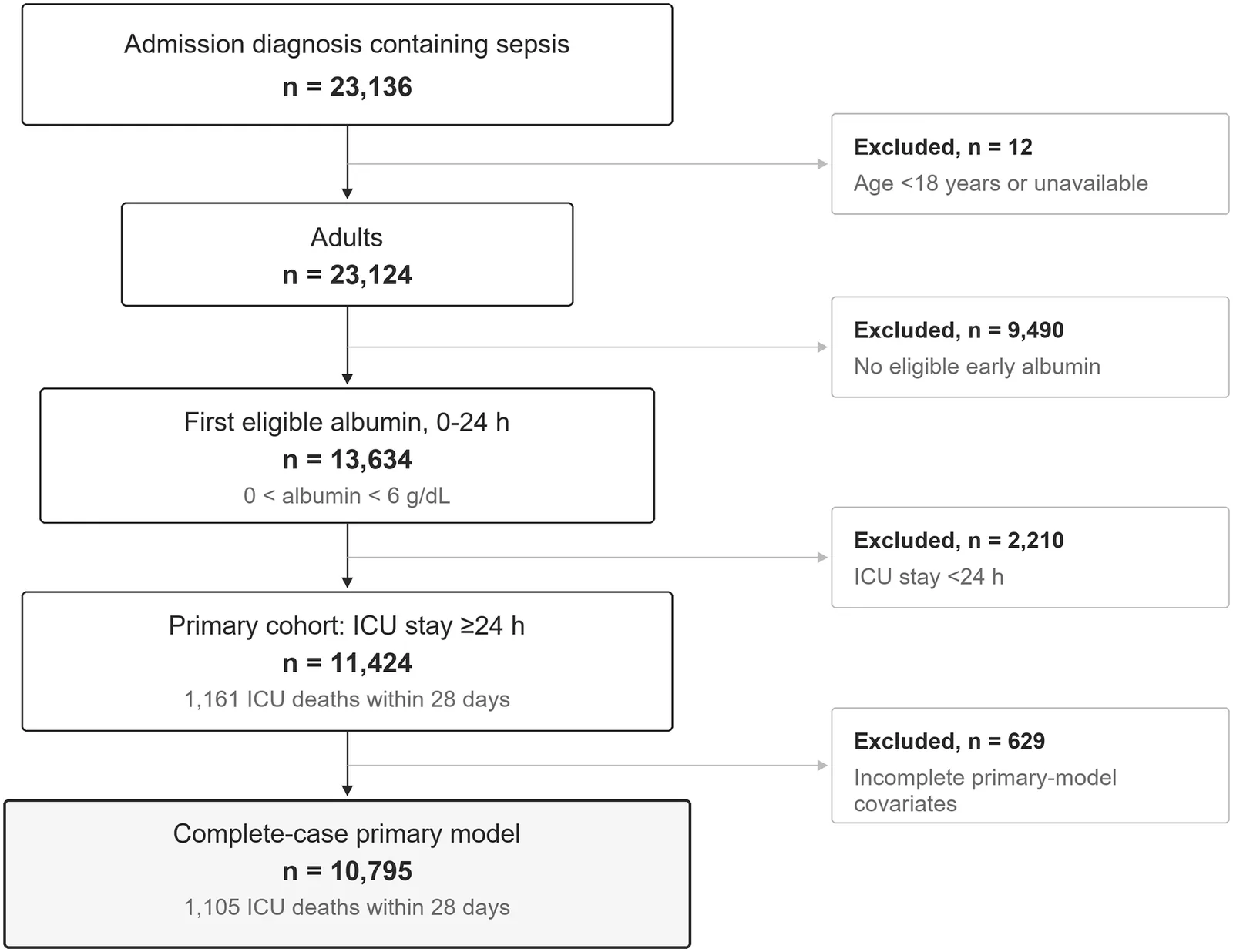
Cohort flow diagram. The cohort was reconstructed from original eICU-CRD v2.0 tables. The parent cohort comprised ICU stays with admission-diagnosis text containing sepsis. The primary cohort required adult age, the first valid serum albumin measurement from 0 to 24 h after ICU admission and before ICU discharge, and an ICU stay of at least 24 h. The complete-case primary association model included 10,795 stays and 1,105 ICU deaths within 28 days.

The primary endpoint was death during the ICU stay within 28 days after ICU admission. Outcome status and time were derived from ICU discharge status and offsets in the patient table. Patients discharged alive and those still alive in the ICU at day 28 did not meet the primary endpoint. In time-to-event analyses, follow-up ended at day 28; discharge alive was treated as censoring in cause-specific Cox models and as a competing event in Fine-Gray models (19–21).

### Covariates

The primary adjustment set comprised age, sex, race/ethnicity, admission weight, hospital discharge year, and recorded comorbidities: AIDS, hepatic failure, lymphoma, metastatic cancer, leukemia, immunosuppression, cirrhosis, and diabetes. A severity-expanded model additionally included APACHE score, mechanical ventilation, dialysis, mean arterial pressure, creatinine, bilirubin, and lactate. This analysis examined how the estimate changed after accounting for early physiological disturbance and organ support.

We considered fluid exposure because hemodilution can influence measured albumin concentration. Twenty-four-hour net fluid balance was summarized but was not included in the primary model because this interval could extend beyond the blood draw and pre-ICU resuscitation was incompletely captured. Entries for albumin in the medication table reflected orders; timing, dose, and completion of administration could not be established. Positive fluid balance is itself associated with adverse outcomes in sepsis (22).

### Statistical analysis

Baseline characteristics were summarized by albumin tertile as median [interquartile range] or number (percentage); variable-specific missingness is shown in Table 1. Multivariable logistic regression estimated odds ratios (ORs) for ICU death within 28 days per 1 g/dL higher albumin concentration, using hospital-clustered robust standard errors. Tertile analyses described the association categorically.

**Table 1:** Baseline characteristics by early albumin tertile.

Characteristic	Missing, *n* (%)	Low (0.6–2.2; *n* = 4,064)	Middle (2.3–2.8; *n* = 4,172)	High (2.9–5.0; *n* = 3,188)	Overall (*n* = 11,424)
Albumin, g/dL	0 (0.0%)	1.9 [1.7, 2.1]	2.5 [2.4, 2.7]	3.2 [3.0, 3.4]	2.5 [2.1, 2.9]
Age, years	0 (0.0%)	66.0 [55.0, 76.0]	68.0 [57.0, 79.0]	66.0 [54.0, 78.0]	67.0 [56.0, 78.0]
Admission weight, kg	211 (1.8%)	74.6 [61.2, 91.0]	77.2 [64.5, 95.3]	81.0 [67.0, 99.8]	77.1 [63.7, 95.3]
28-day ICU death	0 (0.0%)	586 (14.4%)	350 (8.4%)	225 (7.1%)	1,161 (10.2%)
Female sex	0 (0.0%)	2,020 (49.7%)	2,045 (49.0%)	1,503 (47.1%)	5,568 (48.7%)
Caucasian race/ethnicity	0 (0.0%)	3,059 (75.3%)	3,328 (79.8%)	2,469 (77.4%)	8,856 (77.5%)
APACHE score	1,513 (13.2%)	78.0 [63.0, 97.0]	70.0 [55.0, 86.0]	63.0 [49.0, 81.0]	71.0 [55.0, 89.0]
Mechanical ventilation	426 (3.7%)	1,211 (31.1%)	1,189 (29.6%)	910 (29.5%)	3,310 (30.1%)
Dialysis	426 (3.7%)	202 (5.2%)	201 (5.0%)	217 (7.0%)	620 (5.6%)
Mean blood pressure, mmHg	426 (3.7%)	54.0 [46.0, 70.0]	55.0 [47.0, 77.0]	60.0 [49.0, 124.0]	56.0 [47.0, 112.0]
Creatinine, mg/dL	426 (3.7%)	1.4 [0.7, 2.4]	1.3 [0.8, 2.4]	1.3 [0.8, 2.4]	1.3 [0.7, 2.4]
Bilirubin, mg/dL	426 (3.7%)	0.6 [0.3, 1.3]	0.6 [0.3, 1.1]	0.6 [0.3, 1.0]	0.6 [0.3, 1.1]
Lactate, mmol/L	3,941 (34.5%)	2.0 [1.2, 3.4]	1.8 [1.1, 3.0]	1.8 [1.2, 2.9]	1.9 [1.2, 3.1]
AIDS	426 (3.7%)	26 (0.7%)	15 (0.4%)	4 (0.1%)	45 (0.4%)
Hepatic failure	426 (3.7%)	137 (3.5%)	93 (2.3%)	67 (2.2%)	297 (2.7%)
Lymphoma	426 (3.7%)	51 (1.3%)	36 (0.9%)	25 (0.8%)	112 (1.0%)
Metastatic cancer	426 (3.7%)	208 (5.3%)	122 (3.0%)	60 (1.9%)	390 (3.5%)
Leukemia	426 (3.7%)	65 (1.7%)	71 (1.8%)	29 (0.9%)	165 (1.5%)
Immunosuppression	426 (3.7%)	259 (6.7%)	241 (6.0%)	134 (4.3%)	634 (5.8%)
Cirrhosis	426 (3.7%)	208 (5.3%)	118 (2.9%)	78 (2.5%)	404 (3.7%)
Diabetes	426 (3.7%)	912 (23.5%)	1,007 (25.0%)	895 (29.0%)	2,814 (25.6%)

Values are median [IQR] or *n* (%). Tertiles are data-derived and ranges are in g/dL. Missingness is shown for the analytic cohort (*n* = 11,424).

We assessed nonlinearity using restricted cubic splines (23) in the same 10,795 complete cases and 1,105 deaths included in the primary adjusted continuous model. Knots were placed at the 5th, 35th, 65th, and 95th percentiles of albumin (1.5, 2.2, 2.7, and 3.5 g/dL). Every complete case contributed to estimation. The curve is displayed between 1.5 and 3.5 g/dL and is referenced to the cohort median of 2.5 g/dL. Joint Wald tests assessed the overall association and nonlinearity.

Because early albumin testing was clinically selective, we modeled the probability of an eligible measurement among all 23,124 adults using demographic characteristics, admission weight, year, comorbidities, APACHE score and its availability, mechanical ventilation, dialysis, lactate measurement and value, ICU stay type, and hospital teaching status, bed-size category, and region. Stabilized inverse-probability-of-measurement weights were trimmed at the 1st and 99th percentiles, and standardized mean differences assessed covariate balance (24). The weighted outcome model used hospital-clustered robust standard errors and targeted both short and longer ICU stays.

The primary analyses used complete records for their respective covariates. Supplementary Tables 1, 2 describe missingness and mortality according to observed status. We repeated the severity-expanded analysis after 20 chained-equation imputations that included albumin, outcome, model covariates, and selected missingness indicators (25). Continuous variables were imputed by iterative regression with posterior sampling, and binary variables were bounded and rounded before estimates were combined with Rubin's rules. Variance inflation factors are reported in Supplementary Table 3.

**Table 2:** Association of early serum albumin with 28-day ICU mortality.

Analysis	Exposure	*N*	Deaths	OR (95% CI)	*P* value
Unadjusted continuous	Per 1 g/dL higher albumin	11,424	1,161	0.54 (0.47–0.62)	<0.001
Primary adjusted continuous	Per 1 g/dL higher albumin	10,795	1,105	0.54 (0.48–0.62)	<0.001
Severity-expanded continuous	Per 1 g/dL higher albumin	6,466	823	0.72 (0.61–0.83)	<0.001
Primary adjusted tertiles	Middle vs low tertile	10,795	1,105	0.54 (0.48–0.61)	<0.001
Primary adjusted tertiles	High vs low tertile	10,795	1,105	0.46 (0.39–0.55)	<0.001
Restricted cubic spline	Overall association test	10,795	1,105	Joint Wald test of all albumin spline terms	<0.001
Restricted cubic spline	Nonlinearity test	10,795	1,105	Knots 1.5, 2.2, 2.7, 3.5 g/dL; reference median 2.5 g/dL	<0.001

Primary model: age, sex, race/ethnicity, admission weight, hospital discharge year, and comorbidities. Severity-expanded model additionally includes APACHE, mechanical ventilation, dialysis, mean blood pressure, creatinine, bilirubin, and lactate; this is a sensitivity analysis. The spline rows report the overall joint Wald test and the test of nonlinear terms.

Additional analyses were restricted to the first ICU stay, included stays shorter than 24 h under the primary-model complete-case requirements, and used cause-specific Cox and Fine-Gray models in the primary complete-case population. Both time-to-event models used the primary adjustment set and hospital-clustered robust standard errors. We explored interactions with hepatic failure, mechanical ventilation, and dialysis without adjustment for multiple comparisons. C-statistic, Brier score, calibration intercept and slope, and 100-bootstrap optimism-corrected estimates are presented descriptively.

Analyses were performed in Python 3.12.13 using pandas 2.3.3, NumPy 2.3.5, statsmodels 0.14.6, SciPy 1.18.1, patsy 1.0.2, scikit-learn 1.9.0, and lifelines 0.30.3. The random seed was 20260826. Tests were two-sided, and estimates are reported with 95% confidence intervals.

## Results

### Study population and baseline characteristics

Of 23,136 ICU stays in the diagnosis-text parent cohort, 23,124 were adult stays. An eligible albumin measurement within 24 h was available for 13,634 adults (59.0%). Excluding 2,210 stays shorter than 24 h left 11,424 stays in the primary cohort, including 1,161 deaths (10.2%). The primary complete-case analysis comprised 10,795 stays and 1,105 deaths (Figure 1).

Adults with and without an eligible albumin measurement differed in demographic and clinical characteristics, availability of APACHE and lactate measurements, organ support, mortality, and hospital characteristics (Supplementary Table 7 and Supplementary Figure 2). After weighting, the maximum absolute standardized mean difference across measurement-model covariates decreased from 0.171 to 0.014. Baseline characteristics by albumin tertile are presented in Table 1. Mortality was highest in the lowest tertile, which also showed greater overall acute physiological derangement.

### Association between albumin and mortality

Each 1 g/dL higher albumin concentration was associated with lower odds of 28-day ICU mortality in the unadjusted analysis (OR 0.54, 95% CI 0.47–0.62; *n* = 11,424) and in the primary adjusted analysis (OR 0.54, 95% CI 0.48–0.62; 10,795 stays and 1,105 deaths). Compared with the lowest tertile, adjusted ORs were 0.54 (95% CI 0.48–0.61) in the middle tertile and 0.46 (95% CI 0.39–0.55) in the highest tertile. After addition of acute severity variables, the continuous association was attenuated (OR 0.72, 95% CI 0.61–0.83; 6,466 stays and 823 deaths) (Table 2).

The restricted cubic spline was fitted in all 10,795 complete cases from the primary model. Both the overall association and the nonlinear component were significant (*P* < 0.001). The largest change in estimated mortality odds occurred across lower albumin concentrations, while confidence intervals widened toward the upper end of the displayed range (Figure 2 and Table 2).

**Figure 2:**
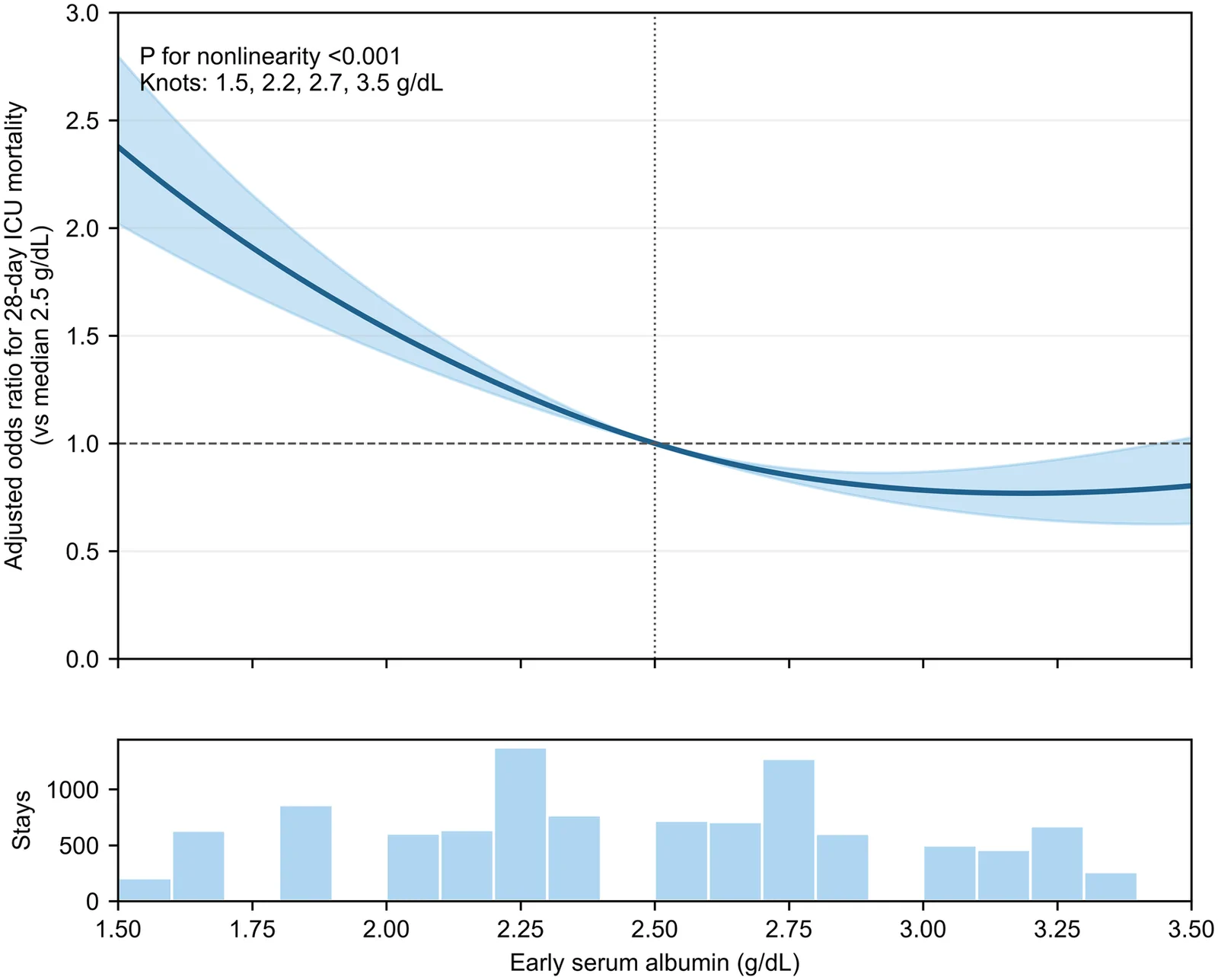
Restricted cubic spline association between early serum albumin and 28-day ICU mortality. The spline was fitted in all 10,795 complete cases (1,105 deaths) used in the primary adjusted model, with knots at 1.5, 2.2, 2.7, and 3.5 g/dL and hospital-clustered robust standard errors. The curve is displayed over 1.5-3.5 g/dL and referenced to the cohort median of 2.5 g/dL; all complete cases contributed to estimation. Overall association and nonlinearity were both *P* < 0.001. The shaded band represents the 95% confidence interval.

### Sensitivity and supplementary analyses

The direction of the association was similar with 6-h (OR 0.52, 95% CI 0.44–0.63), 12-h (OR 0.52, 95% CI 0.44–0.60), and 48-h (OR 0.55, 95% CI 0.48–0.62) measurement windows and after restriction to first ICU stays (OR 0.53, 95% CI 0.46–0.60). The inverse-probability-weighted analysis targeted all 23,124 adults. Of these, 13,634 had an eligible measurement; 12,857 complete cases with 1,456 deaths contributed to the weighted outcome model (OR 0.48, 95% CI 0.42–0.54) (Table 3 and Supplementary Figure 2).

**Table 3:** Sensitivity analyses.

Analysis	Measure	*N*	Deaths	Estimate (95% CI)	*P* value	Model
Primary 0–24 h association	OR	10,795	1,105	0.54 (0.48–0.62)	<0.001	A
0–6 h albumin window	OR	4,678	523	0.52 (0.44–0.63)	<0.001	A
0–12 h albumin window	OR	7,526	804	0.52 (0.44–0.60)	<0.001	A
0–48 h albumin window	OR	12,210	1,240	0.55 (0.48–0.62)	<0.001	A
Including ICU stays <24 h	OR	12,857	1,456	0.49 (0.43–0.55)	<0.001	A
First ICU stay only	OR	9,591	969	0.53 (0.46–0.60)	<0.001	A
IPW for albumin availability	OR	12,857	1,456	0.48 (0.42–0.54)	<0.001	B
Cause-specific Cox model	HR	10,795	1,105	0.73 (0.64–0.82)	<0.001	A
Fine-Gray competing-risk model	sHR	10,795	1,105	0.56 (0.47–0.66)	<0.001	A

Model A adjusted for age, sex, race/ethnicity, admission weight, hospital discharge year, and recorded comorbidities, with hospital-clustered robust standard errors. Model B used stabilized inverse-probability-of-measurement weights derived from demographic, comorbidity, acute-severity availability, ICU, and hospital variables; weights were trimmed at the 1st and 99th percentiles, and the outcome model used hospital-clustered robust standard errors. The IPW target population included ICU stays shorter than 24 h. Cox and Fine-Gray models used Model A in the primary complete-case population. OR, odds ratio; HR, hazard ratio; sHR, subdistribution hazard ratio.

.

Among the 2,210 stays shorter than 24 h, 369 deaths occurred. When these stays were included, the analysis contained 13,634 albumin-eligible stays and 12,857 complete cases with 1,456 deaths; the OR was 0.49 (95% CI 0.43–0.55). In the primary complete-case population, the cause-specific Cox model yielded an HR of 0.73 (95% CI 0.64–0.82) and the Fine-Gray model yielded an sHR of 0.56 (95% CI 0.47–0.66) (Table 3, Figure 3, Supplementary Table 8, and Supplementary Figure 4).

**Figure 3:**
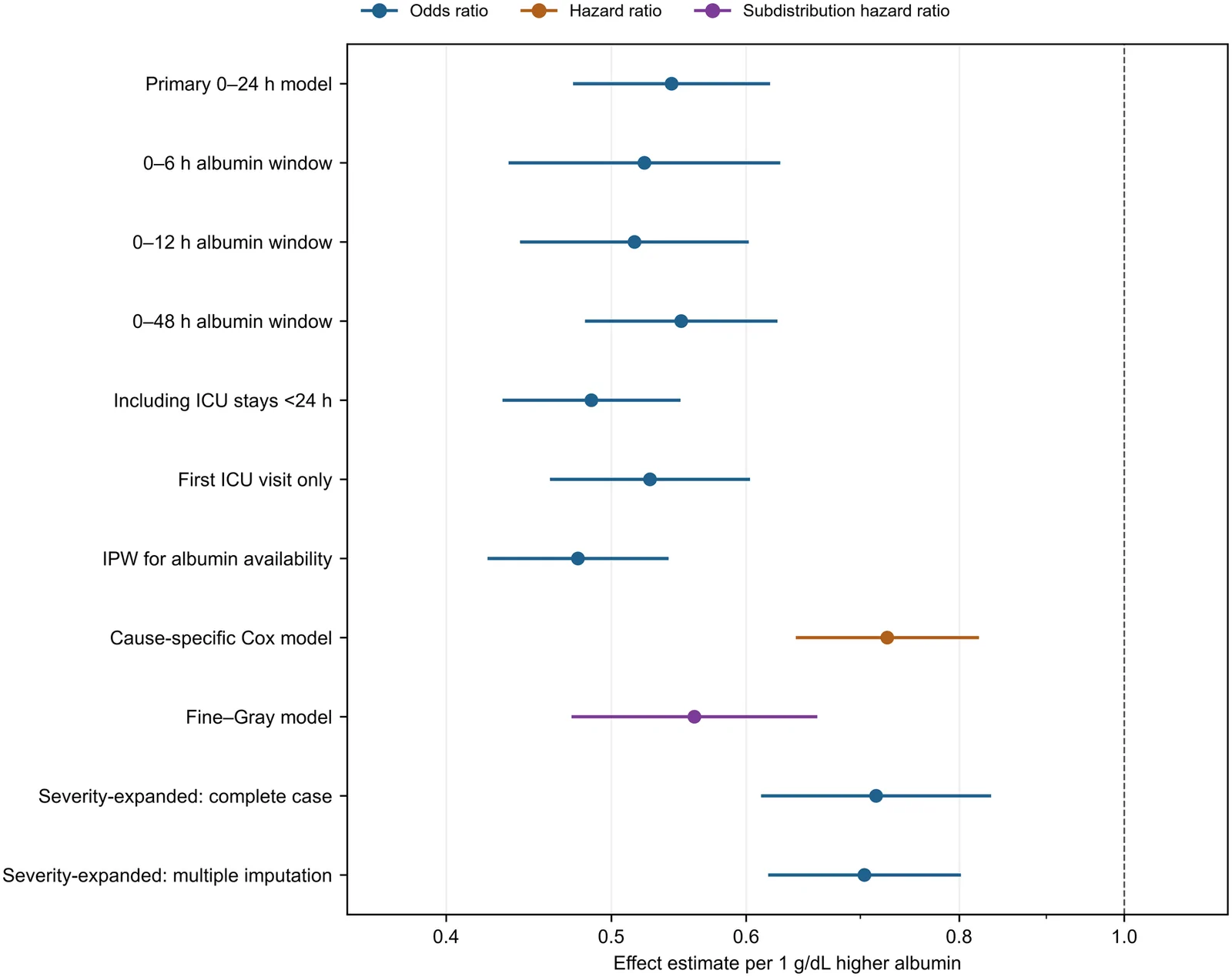
Robustness analyses for the association between early serum albumin and 28-day ICU mortality. Effect estimates are per 1 g/dL higher albumin. Blue symbols denote odds ratios, brown symbols hazard ratios, and purple symbols subdistribution hazard ratios; the three measures estimate different quantities. Horizontal lines show 95% confidence intervals. Model populations and adjustment sets are reported in Tables 2, 3 and Supplementary Table 2.

The interaction with hepatic failure had a nominal *P* value of 0.014, although estimates in this small subgroup were imprecise and interaction analyses were not adjusted for multiple comparisons (Supplementary Table 4 and Supplementary Figure 3). Evidence for interaction with mechanical ventilation or dialysis was weaker. Addition of albumin to the baseline covariate model increased the apparent C-statistic from 0.607 to 0.650 and reduced the apparent Brier score from 0.0905 to 0.0893; bootstrap-corrected performance estimates are shown in Supplementary Table 6.

## Discussion

In this multicenter cohort, lower serum albumin measured early after ICU admission was associated with higher 28-day ICU mortality. The association was nonlinear, with the greatest change in estimated mortality odds across the lower part of the observed concentration range. The direction of the association was maintained when the measurement window was varied, when short stays were included, and when repeated admissions, missing data, selective testing, and time to death were handled differently. The estimate became smaller after acute severity and organ-support variables were added, indicating substantial overlap between albumin concentration and the physiological disturbance present early in critical illness.

Earlier observational studies have related albumin measured at admission, later during admission, or serially over time to mortality in sepsis (10–14). Differences in recruitment setting, blood-sampling time, endpoint definition, and adjustment for illness severity limit direct comparison of effect sizes. The present study concentrated on the first eligible result during the first 24 h of ICU care and evaluated the association across the observed range rather than at a single cut point. Calculated globulin has also been associated with mortality in an eICU cohort (26). Because calculated globulin is derived from total protein and albumin, it represents a different protein-related measure; together, these studies indicate that disturbances in circulating proteins accompany adverse outcomes during critical illness.

Several linked processes could contribute to the observed association. Sepsis-associated endothelial activation and glycocalyx injury increase vascular permeability and favor movement of albumin from the intravascular to the interstitial compartment (2–4, 27–30). Reduced hepatic synthesis and dilution during resuscitation can further lower the circulating concentration. Chronic liver or kidney disease, nutritional status, and other pre-existing illnesses also influence albumin before the acute event. A low concentration early in ICU care may thus arise when acute vascular injury is superimposed on limited physiological reserve. The attenuation in the severity-expanded model is consistent with this clinical overlap, although some acute severity variables may also lie on the pathway between early illness and albumin concentration.

Early albumin may add useful context to the assessment provided by haemodynamic findings, organ dysfunction, organ support, comorbidity, and fluid exposure. The modest improvement in descriptive discrimination supports its use as one component of a broader clinical assessment. The curve was most informative at lower concentrations, but clinically actionable cut points require evaluation in independent contemporary cohorts. Decisions about albumin administration should continue to be guided by randomized trials, evidence syntheses, and guidelines (5–9, 31–34).

Several limitations should be considered. The cohort was defined from admission diagnosis text and did not permit application of the infection-plus-acute-organ-dysfunction criteria used in contemporary sepsis definitions. Only 59.0% of eligible adults had an early albumin measurement, and patients who were tested differed from those who were not in clinical and hospital characteristics. Weighting improved balance in recorded determinants of testing, but clinician decisions that were not captured in the database and missing-not-at-random mechanisms may still have influenced the association. The primary 24-h stay requirement also excluded patients who died early and those discharged rapidly, although inclusion of short stays produced a similar estimate.

A single early result cannot characterize albumin trajectories, pre-ICU fluid exposure, intravascular volume, or later albumin administration. The 24-h fluid-balance interval could include events after the blood draw, and medication orders did not establish actual administration. Residual confounding is therefore likely. It is also possible that adjustment for acute severity variables partly conditioned on processes related to the albumin measurement itself. Missing acute measurements remained substantial despite multiple imputation. Finally, eICU-CRD reflects United States ICU practice in 2014–2015, and the findings have not been assessed in an independent cohort.

Future cohorts with prespecified infection and organ-dysfunction definitions should examine early and serial albumin measurements alongside fluid exposure and confirmed albumin administration. Independent studies are needed to determine whether particular concentration ranges improve clinical risk assessment. In this cohort, early serum albumin was nonlinearly associated with 28-day ICU mortality and may provide complementary prognostic information, especially at lower concentrations. Whether albumin-directed treatment improves outcomes requires prospective interventional study.

## Conclusion

Among adults admitted with a diagnosis containing sepsis, lower early serum albumin was nonlinearly associated with higher 28-day ICU mortality, with the greatest change in estimated mortality odds across the lower observed range. Early albumin may provide complementary information for clinical risk assessment. Independent contemporary cohorts should evaluate clinically actionable concentration ranges, and interventional studies are required to determine whether albumin administration improves outcomes.

## Data Availability

Data analyzed in this study are available in the eICU Collaborative Research Database v2.0 on PhysioNet (https://physionet.org/content/eicu-crd/2.0/). Access is available to credentialed users who complete the required training and sign the applicable data use agreement.
