# Supplementary material for: Nonlinear association of early serum albumin with 28-day ICU mortality in adults with an admission diagnosis of sepsis: a multicenter eICU cohort study
Source: Front Med (Lausanne). 2026 Sep 29;13:1954894. doi: 10.3389/fmed.2026.1954894 (PMC13658449; doi:10.3389/fmed.2026.1954894)

Supplementary Material

Supplementary Table 1. Missingness and mortality according to covariate availability.

Variable	Missing, n (%)	Mortality if missing, %	Mortality if observed, %
Admission weight	211 (1.8%)	9.5	10.2
AIDS	426 (3.7%)	8.5	10.2
Hepatic failure	426 (3.7%)	8.5	10.2
Lymphoma	426 (3.7%)	8.5	10.2
Metastatic cancer	426 (3.7%)	8.5	10.2
Leukemia	426 (3.7%)	8.5	10.2
Immunosuppression	426 (3.7%)	8.5	10.2
Cirrhosis	426 (3.7%)	8.5	10.2
Diabetes	426 (3.7%)	8.5	10.2
APACHE score	1,513 (13.2%)	7.6	10.6
Mechanical ventilation	426 (3.7%)	8.5	10.2
Dialysis	426 (3.7%)	8.5	10.2
Mean blood pressure	426 (3.7%)	8.5	10.2
Creatinine	426 (3.7%)	8.5	10.2
Bilirubin	426 (3.7%)	8.5	10.2
Lactate	3,941 (34.5%)	6.1	12.3

Note. Percentages use the primary analytic cohort (n=11,424). Mortality percentages compare records with missing versus observed values; they are descriptive and do not establish the direction of missing-data bias.

Supplementary Table 2. Complete-case and multiple-imputation severity-expanded analyses.

Analysis	N	Deaths	OR (95% CI)	P value	Imputations	Within variance	Between variance
Severity-expanded complete case	6,466	823	0.72 (0.61-0.83)	<0.001			
Severity-expanded multiple imputation	11,424	1,161	0.70 (0.62-0.80)	<0.001	20	0.0042	0.0001

Note. Odds ratios are per 1 g/dL higher albumin. Twenty imputations were combined using Rubin's rules. The severity-expanded model included APACHE score, mechanical ventilation, dialysis, mean blood pressure, creatinine, bilirubin, and lactate in addition to primary-model covariates.

Supplementary Table 3. Variance inflation factors for the primary adjusted model.

Model term	VIF
Male sex	2.16
Asian race/ethnicity	1.16
Caucasian race/ethnicity	8.13
Hispanic race/ethnicity	1.30
Native American race/ethnicity	1.09
Other/unknown race/ethnicity	1.46
race: Unknown	1.06
hospitaldischargeyear: 2015	2.16
age_numeric	12.47
admissionweight	9.51
aids	1.01
hepaticfailure	1.54
lymphoma	1.04
metastaticcancer	1.13
leukemia	1.05
immunosuppression	1.21
cirrhosis	1.57
diabetes	1.41
albumin_0_24h_gdl	13.55

Note. VIF, variance inflation factor. Categorical-variable coefficients are displayed separately. Values reflect the primary complete-case design matrix.

Supplementary Table 4. Exploratory interaction analyses.

Modifier	N	Deaths	Interaction ratio (95% CI)	P for interaction
Hepatic failure	10,795	1,105	1.93 (1.14-3.27)	0.014
Mechanical ventilation	10,795	1,105	0.94 (0.75-1.18)	0.617
Dialysis	10,795	1,105	1.43 (0.85-2.43)	0.180

Note. Interaction ratios compare albumin effect estimates across strata. No multiple-comparison adjustment was applied; all interaction findings are exploratory.

Supplementary Table 5. Variable definitions and provenance.

Variable	eICU table	Source field(s)	Operational definition	Analysis role/limitation
Cohort diagnosis	admissionDx	admitdxname, admitdxtext, admitdxpath	Admission diagnosis text containing 'sepsis'	No Sepsis-3 claim
Age	patient	age	Parsed age; adults aged >=18 years	Unavailable age excluded
Sex	patient	gender	Recorded sex	Categorical
Race/ethnicity	patient	ethnicity	Recorded eICU category	Categorical
Hospital	patient	hospitalid	Hospital identifier	Cluster-robust SEs
Admission weight	patient	admissionweight	Recorded admission weight, kg	Primary covariate
ICU duration	patient	unitdischargeoffset	Minutes from ICU admission to discharge	Primary cohort >=24 h
28-day ICU death	patient	unitdischargestatus; unitdischargeoffset	Death in ICU within 28 days of ICU admission	Outcome
Early albumin	lab	labname; labresult; labresultoffset	First albumin at 0–24 h and before ICU discharge; 0 <= value <6 g/dL	Exposure, g/dL
Lactate	lab	labname; labresult; labresultoffset	Available early laboratory lactate	Severity sensitivity
APACHE score	apachePatientResult	apachescore	Recorded APACHE score	Severity sensitivity
Mechanical ventilation	apacheApsVar	vent	Recorded APACHE ventilation indicator	Severity sensitivity
Dialysis	apacheApsVar	dialysis	Recorded APACHE dialysis indicator	Severity sensitivity
Physiologic/laboratory severity	apacheApsVar	meanbp; creatinine; bilirubin	Recorded APACHE physiology variables	Severity sensitivity
Comorbidities	apachePredVar	aids; hepaticfailure; lymphoma; metastaticcancer; leukemia; immunosuppression; cirrhosis; diabetes	Recorded APACHE comorbidity indicators	Primary covariates
Albumin order/fluid records	medication; infusionDrug; intakeOutput	drug/order and intake-output fields	Coverage and timing audit only	Not treated as verified administration or baseline confounding

Note. The cohort was defined by admission diagnosis text containing 'sepsis'. The available tables did not support a reliable infection-plus-acute-SOFA-increase algorithm; the cohort is therefore not described as Sepsis-3. Original eICU data remain subject to PhysioNet access controls.

Supplementary Table 6. Descriptive model performance.

Model	N	Deaths	C-statistic apparent / corrected	Brier score apparent / corrected	Calibration intercept apparent / corrected	Calibration slope apparent / corrected	Bootstrap repetitions
Base covariates	10,795	1,105	0.6068 / 0.5954	0.0905 / 0.0911	0.0033 / -0.2156	1.0016 / 0.8967	100
Base covariates plus albumin	10,795	1,105	0.6495 / 0.6409	0.0893 / 0.0900	-0.0051 / -0.1453	0.9972 / 0.9278	100

Note. Performance measures are descriptive, not an external validation or a clinical prediction-tool assessment. Optimism correction used bootstrap resampling.

Supplementary Table 7. Characteristics according to early albumin availability.

Characteristic	Albumin observed (n=13,634)	Albumin not observed (n=9,490)
Age, years	67.0 [56.0, 78.0]	68.0 [57.0, 79.0]
Female sex	6,699 (49.1%)	4,700 (49.5%)
APACHE score	70.0 [54.0, 88.0]; missing 1,958 (14.4%)	58.0 [44.0, 74.0]; missing 1,758 (18.5%)
Mechanical ventilation	3,714 (28.3%); missing 506 (3.7%)	2,109 (23.8%); missing 633 (6.7%)
Dialysis	730 (5.6%); missing 506 (3.7%)	419 (4.7%); missing 633 (6.7%)
Lactate measured	8,810 (64.6%)	4,269 (45.0%)
Lactate, mmol/L (among measured)	1.9 [1.2, 3.2]	1.7 [1.1, 2.7]
28-day ICU death	1,530 (11.2%)	874 (9.2%)
Hepatic failure	352 (2.7%)	114 (1.3%)
Metastatic cancer	466 (3.5%)	289 (3.3%)
Immunosuppression	767 (5.8%)	484 (5.5%)
Cirrhosis	473 (3.6%)	142 (1.6%)
Diabetes	3,338 (25.4%)	2,138 (24.1%)
Unique hospitals	196	199
Teaching hospitals	2,837 (20.8%)	2,126 (22.4%)
Hospital bed-size category recorded	11,964 (87.8%)	8,462 (89.2%)
Hospital region recorded	12,381 (90.8%)	8,936 (94.2%)

Note. Values are median [IQR] or n (%). Availability was clinically selected; standardized differences are shown in Supplementary Figure 2. IPW was used only as a sensitivity analysis.

Supplementary Table 8. Audit of ICU stays shorter than 24 hours.

Characteristic	<24 h	>=24 h (primary cohort)
ICU stays, n	2,210	11,424
28-day ICU deaths, n (%)	369 (16.7%)	1,161 (10.2%)
Early albumin, g/dL, median [IQR]	2.6 [2.1, 3.0]	2.5 [2.1, 2.9]
Age, years, median [IQR]	66.0 [55.0, 78.0]	67.0 [56.0, 78.0]
First albumin measurement, min, median [IQR]	348.5 [127.0, 591.0]	454.0 [150.8, 806.0]
Complete-case sensitivity model including both stay-duration groups	OR 0.49 (0.43-0.55); n=12,857; deaths=1,456	Primary model shown in Table 2

Note. Values are descriptive. The 11,424 stays in the primary cohort and 2,210 stays shorter than 24 hours formed a combined albumin-eligible cohort of 13,634 stays. Applying the primary-model complete-case requirements yielded 12,857 stays and 1,456 deaths for the short-stay sensitivity regression. These comparisons do not determine the direction of selection bias.

Supplementary Figure 1. Timing and window coverage of early albumin measurement. (A) Distribution of the first valid albumin measurement recorded from ICU admission through 24 h and before ICU discharge. (B) Number of adult admission-diagnosis-defined sepsis stays with a valid albumin measurement and deaths within each measurement window. The primary analysis used the 0–24 h window and the first valid measurement before ICU discharge.

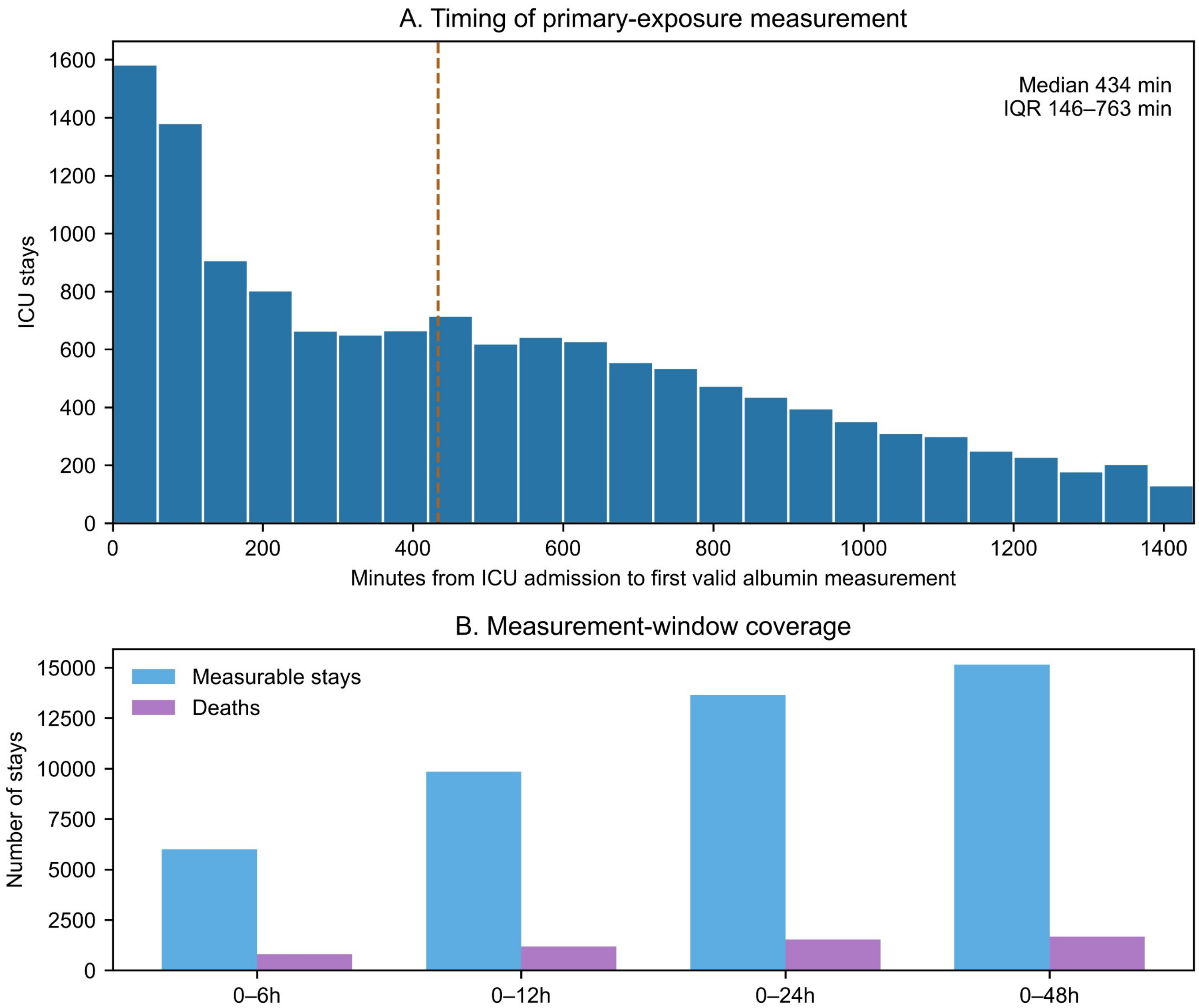

Supplementary Figure 2. Albumin availability and inverse-probability-weighting audit among 23,124 adults with an admission diagnosis containing sepsis. (A) Standardized differences directly compare adults with versus without a valid 0–24 h albumin measurement before ICU discharge. (B) Absolute standardized differences compare the albumin-observed sample with the full adult target population, before and after applying stabilized, 1st/99th-percentile-trimmed weights from the expanded measurement-selection model. The maximum absolute standardized difference across all 56 encoded model terms decreased from 0.171 before weighting to 0.014 after weighting; no term exceeded 0.10 after weighting. Because panels A and B use different comparison populations, their numerical standardized differences are not expected to be identical. This measured-variable analysis does not establish a missing-at-random mechanism or eliminate unmeasured selection bias.

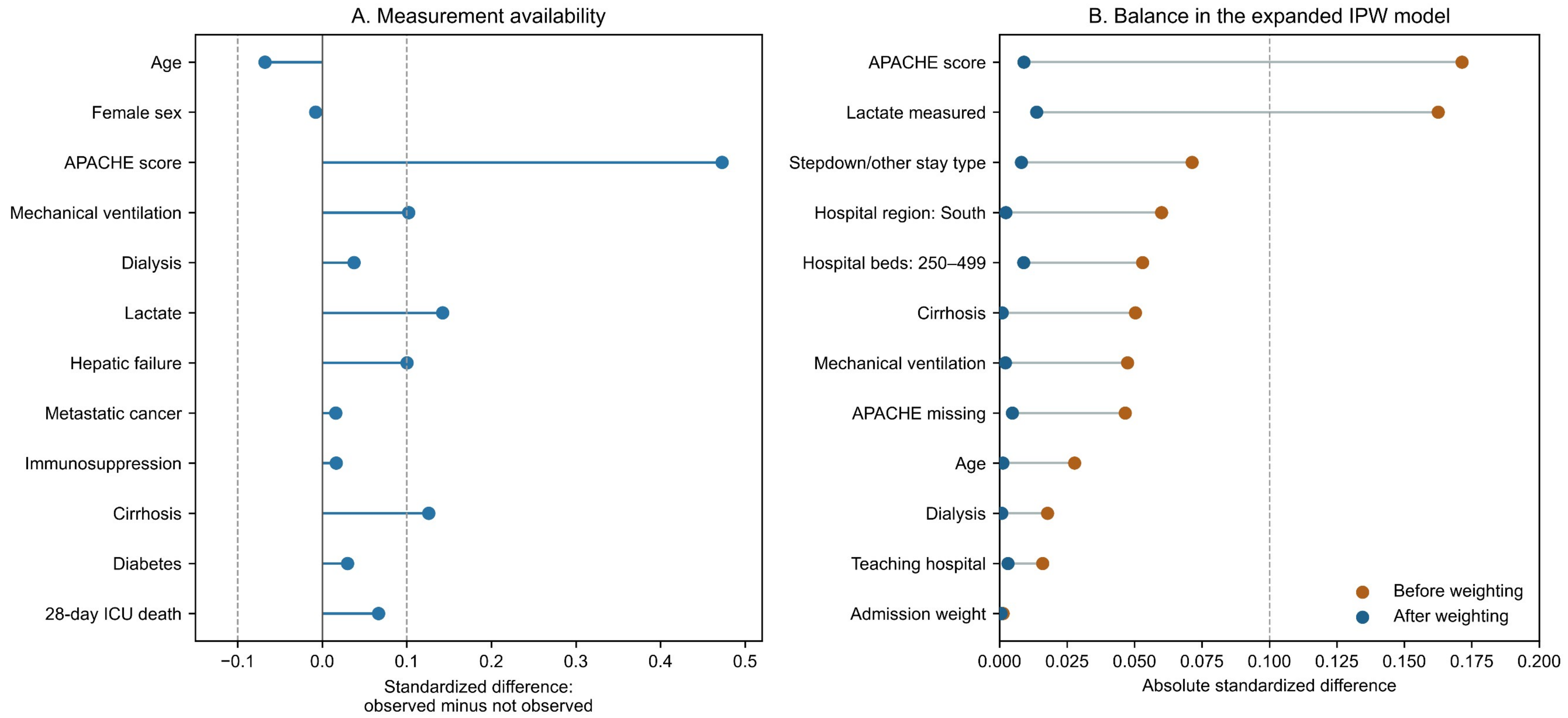

Supplementary Figure 3. Exploratory interaction analyses for the association of early albumin with 28-day ICU mortality. Points are interaction ratios and bars are 95% confidence intervals. No multiple-comparison adjustment was applied. The nominal hepatic-failure interaction (P=0.014) is exploratory and should not be interpreted as a definitive subgroup finding.

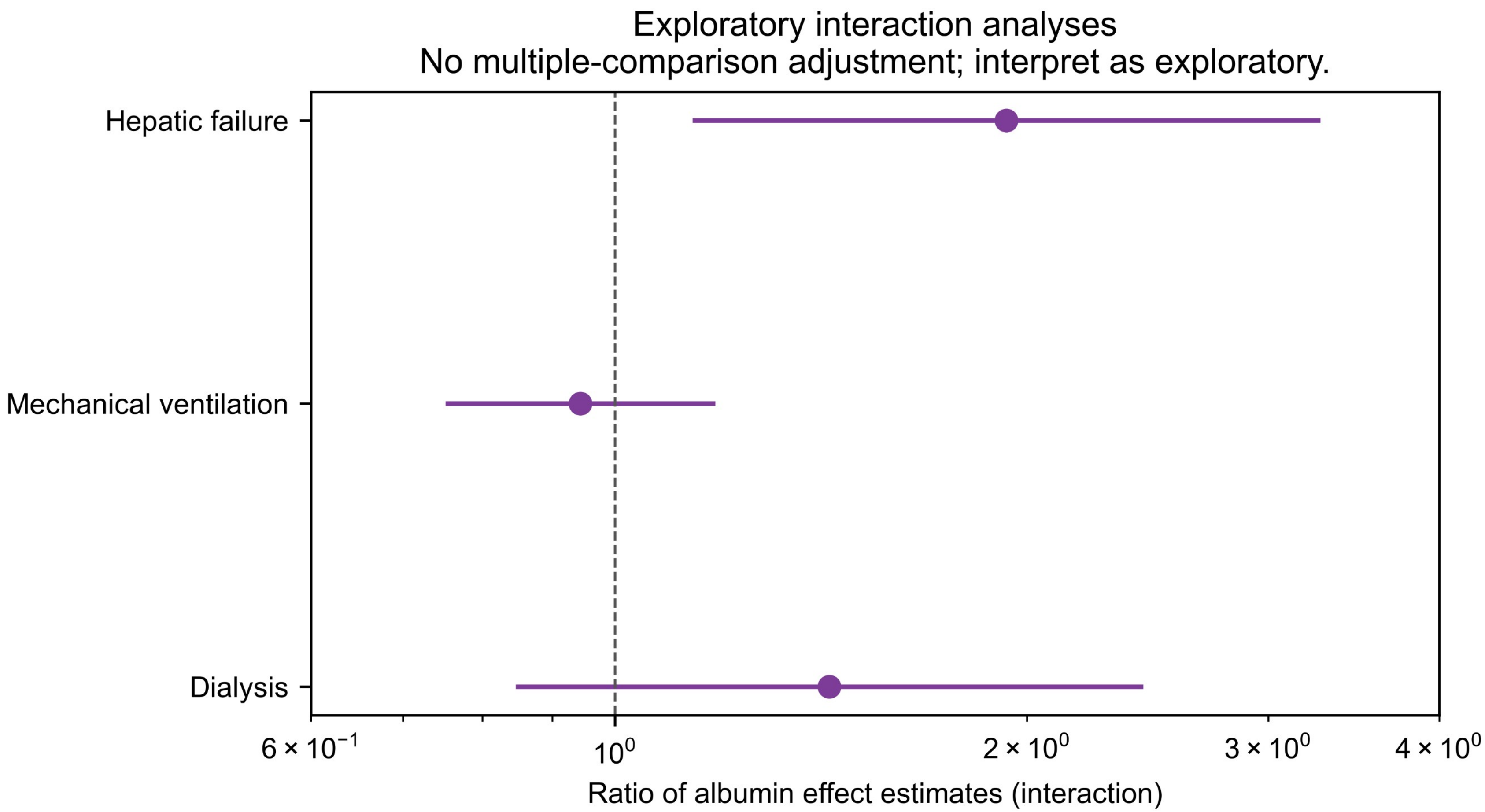

Supplementary Figure 4. Short ICU-stay selection audit among adults with a valid albumin measurement before ICU discharge. Stays shorter than 24 h were excluded from the primary analytic cohort but were included in a sensitivity analysis. This descriptive comparison does not determine the direction of selection bias.

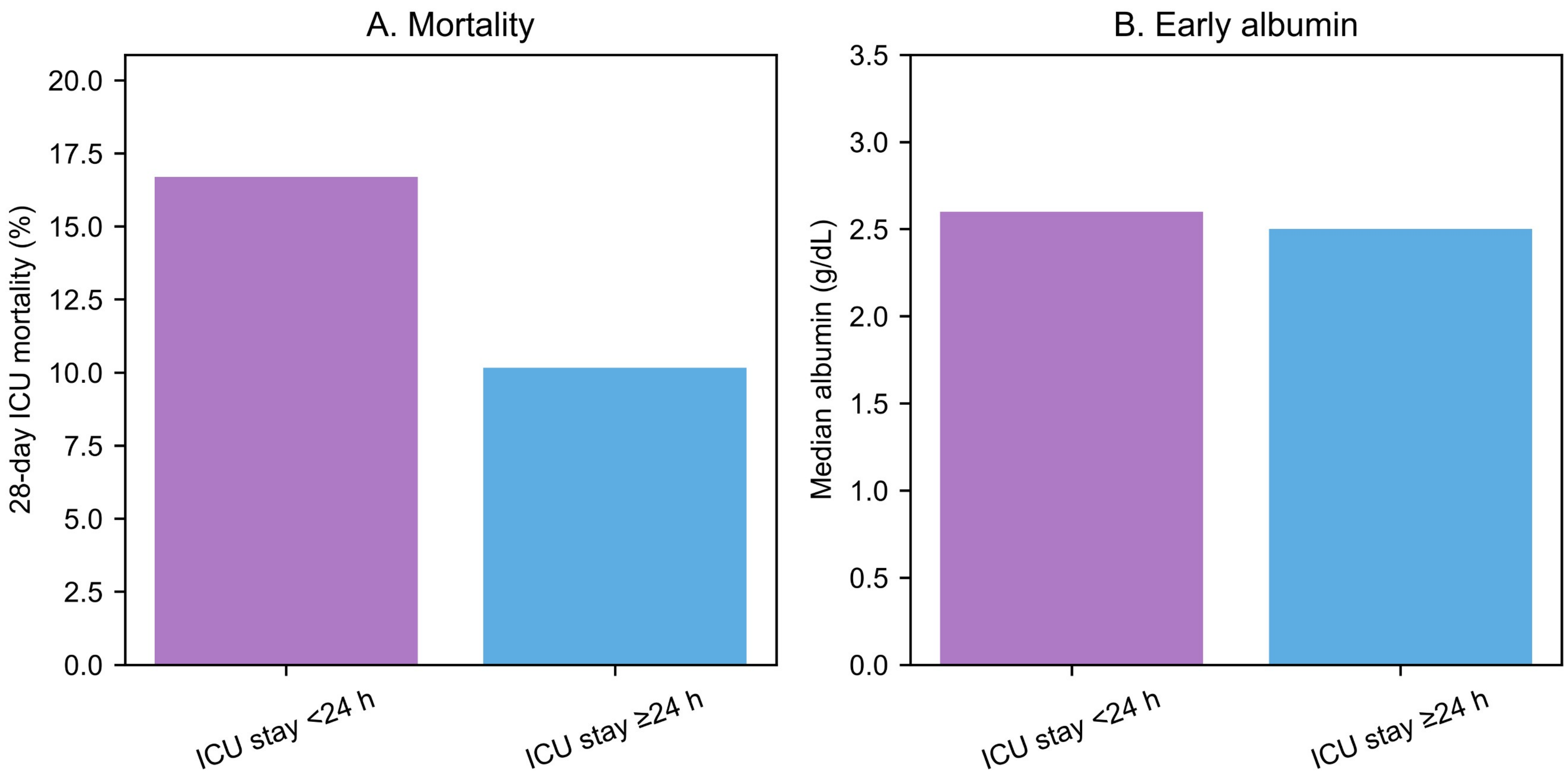

Supplement: Supplementary file 1 [file Datasheet1.pdf]
